# Long-Term Follow-Up of Proteinuria and Estimated Glomerular Filtration Rate in HIV-Infected Patients with Tubular Proteinuria

**DOI:** 10.1371/journal.pone.0142491

**Published:** 2015-11-16

**Authors:** Hélène Peyriere, Amandine Cournil, Marie-Laure Casanova, Stéphanie Badiou, Jean-Paul Cristol, Jacques Reynes

**Affiliations:** 1 UMI 233/INSERM U1175, Institut de Recherche pour le Développement, Université Montpellier, Montpellier, France; 2 Centre Hospitalier Universitaire, Département des Maladies Infectieuses et Tropicales, Montpellier, France; 3 Centre Hospitalier Universitaire, Département de Biochimie, Montpellier, France; 4 UMR 204 NUTRIPASS, Université Montpellier, Montpellier, France; Temple University, UNITED STATES

## Abstract

**Objective:**

The objective of this prospective observational study was to describe the evolution of tubular proteinuria detected in HIV-infected patients, and to evaluate the impact of tenofovir disoproxil fumarate (TDF) discontinuation.

**Methods:**

Proteinuria and estimated glomerular filtration rate (eGFR) were followed during a median duration of 32 months, in 81 HIV-infected patients with tubular proteinuria and eGFR ≥ 60 ml/min/1.73 m^2^ (determined using the Chronic Kidney Disease Epidemiology (CKD-EPI) Collaboration equation). Tubular proteinuria was defined by urine protein to creatinine ratio (uPCR) ≥200 mg/g and albumin to protein ratio (uAPR) <0.4.

**Results:**

Twenty per cent of patients had persistence of tubular proteinuria: TDF continuation was the main factor associated with this persistence [OR 9.0; 95%CI: 1.9–41.4; p = 0.01]. Among the 23 patients who discontinued TDF, uPCR returned below the threshold of 200 mg/g in 11 patients. Overall, eGFR decreased with a mean rate of decline of 3.8 ml/min/1.73m^2^/year. The decline in eGFR was lesser after discontinuation of TDF (5.8 ml/min/1.73m^2^/year during TDF exposure versus 3 ml/min/1.73m^2^/year after TDF discontinuation; p = 0.01).

**Conclusions:**

The continuation of TDF was the main factor associated with the persistence of proteinuria. Moreover, proteinuria was normalized in only half of the patients who discontinued TDF. The clinical significance of TDF-related low level of proteinuria as a factor associated with renal disease progression and bone loss remains poorly understood.

## Introduction

Tenofovir disoproxil fumarate (TDF) has been associated with a wide variety of renal damages, including the well-documented tubular proximal dysfunction [1,2], but also a decrease in estimated glomerular filtration rate (eGFR) [3], chronic kidney disease (CKD) [4], and increased mortality [5]. Proteinuria can precede alteration of eGFR and represents an early marker of renal damage [6]. This is why monitoring these two parameters is important in a population at risk of developing changes in renal function.

Few data are available concerning the reversibility of such subclinical abnormalities when TDF is stopped or their worsening when TDF is continued. Most available data concerned the reversibility of eGFR decline after the discontinuation of TDF, which occurred in 42% to 61% of patients [7–9]. Data on the reversibility of proteinuria upon the discontinuation of TDF are more limited, and the results are discordant [4,10]. To our knowledge, there are no published data on the follow-up of tubular proteinuria.

A cross-sectional study conducted between November 2010 and April 2011, on 1158 HIV-infected adults followed at the university hospital of Montpellier (France) and having an eGFR greater than 60 ml/min/1.73m^2^, detected tubular proteinuria in 107 of these patients (9.2%), and identified associated factors including TDF [11].

In continuation of the previous study [11], this longitudinal study aimed to describe the evolution of tubular proteinuria and eGFR and evaluate the impact of TDF discontinuation.

## Material and Methods

Among the patients with tubular proteinuria at baseline, only patients who had at least two further follow-up visits with urinary biological evaluation were included in the present analysis. The last follow-up visit was in October 2013.

Biological and clinical data were collected from the patient medical file (e-NADIS). Each patient received the necessary information and gave written consent [12]. Review and approval of our study protocol by an ethic committee was not necessary because the longitudinal monitoring of renal function in these patients was included in the comprehensive management of HIV disease. No blood test was performed for this study and urinary parameters are part of the regular monitoring recommended for all patients.

For each visit with urinary biological evaluation, the following data were collected: age, bodyweight, comorbid conditions (diabetes, arterial hypertension).

During this study in real life conditions, antiretroviral therapy was checked at each visit and therapeutic changes and their reasons were collected.

The biological data collected were enzymatically-determined creatininemia, proteinuria and albuminuria.

Estimated GFR (eGFR) was determined using the Chronic Kidney Disease Epidemiology (CKD-EPI) Collaboration equation [13] allowing determination of eGFR up to stage 1 of CKD [14]. Spot urine protein to creatinine (uPCR), albumin to creatinine (uACR), and albumin to protein (uAPR) ratios were assessed. Proteinuria was defined as uPCR ≥200 mg/g. Tubular proteinuria was defined as uPCR ≥200 mg/g with uAPR <0.4 [11,15].

Three categories of TDF exposure during the follow-up were considered: “TDF continuation” for patients who received TDF during the follow-up without discontinuation; “TDF discontinuation” for patients who received TDF at baseline and stopped it at any time during the follow-up; and “no TDF exposure” for patients who were not exposed to TDF during the follow-up.

### Statistical analysis

Baseline characteristics were compared between TDF exposure groups using chi2 test, analysis of variance or kruskall-wallis test.

Persistence of tubular proteinuria was defined in patients who were followed for at least 2 years, as presence of tubular proteinuria at all visits with an authorized exception. Factors associated with persistence of tubular proteinuria were investigated using multivariate logistic regressions. Mixed-effects linear models were used to examine the TDF exposure group effect on uPCR and eGFR taken as continuous variables. Random intercept and slope were included in the model to account for multiple measurements per individual. Mean levels during the whole follow-up and modification over time (slope) were analyzed. Covariates considered in the different models were age, sex, weight, hypertension, AIDS stage, CD4 count level <200 cells/μL, undetectable viral load, protein to creatinine ratio, previous TDF exposure duration, and boosted protease inhibitor exposure. Baseline values were taken into account. All analyses were adjusted for age and sex and all other covariates associated with the outcome with a P-value <0.20 in univariate analysis were included in the multivariate model. In subjects who discontinued TDF therapy, levels and changes over time were assessed during TDF exposition and after discontinuation using linear mixed models. These analyses were only adjusted for age and sex. Log transformation was applied for uPCR to approach a normal distribution.

## Results

### Characteristics of the patients

Out of 107 patients who presented with a tubular proteinuria at baseline, 81 patients had at least two follow-up visits with urinary biological evaluation and were included in the analysis. Patients with less than two follow-up visits were more likely to have a positive hepatitis C serology but had less frequently reached AIDS stage at baseline. The 81 patients included were mainly men (n = 65; 80%) with a median age of 51 years [Interquartile range (IQR): 46–59]. Thirty-six patients (44%) were at AIDS stage, HIV viral load was inferior to 20 copies/ml in 59 patients (74%), and CD4 cell count was inferior to 200/mm^3^ in 12 patients (15%). In patients with HIV viral load > 20 copies/ml, the median HIV viral load was 68 [IQR: 27;1792] copies/ml.

Concerning the comorbid conditions, 13 patients (16%) had hepatitis C co-infection (anti-HCV+), 10 patients (12%) arterial hypertension and 5 patients (6%) diabetes.

At baseline, 58 patients (71%) received ritonavir boosted protease inhibitors. Eight patients were treated for arterial hypertension: 3 with angiotensin2 receptor antagonists (losartan, candesartan, irbesartan), 4 with ACE inhibitors (name not documented) and 1 with hydrochlorothiazide.

The median duration of follow-up was 32 months [IQR: 29.2–33.3]. The minimum duration of follow-up was 10 months. A total of 736 visits were recorded with a median of 9 visits per patient [IQR: 8–11].

Among the 81 patients, 59 (73%) received antiretroviral therapy containing TDF at baseline. The remaining 22 patients had no TDF at baseline and did not receive TDF during follow-up. Among the 59 patients having TDF at baseline, 28 (47%) stopped it during the follow-up. The median follow-up at the time of TDF discontinuation was 7.5 months [IQR: 0–12.3]. The medical reasons for TDF discontinuation were renal disturbances (eGFR < 90 ml/min/1,73m^2^, persistent proteinuria) for 24 patients (85%), bone disturbances (osteopenia, osteonecrosis) for 2 patients (7%), and virological failure for 2 patients (7%). No Fanconi syndrome was observed.

The demographic characteristics of patients did not differ between TDF groups (Table 1).

**Table 1:** Clinical and Biological characteristics of the 81 patients at baseline, according to TDF exposure.

	TDF discontinuation n = 28	TDF continuation n = 31	No TDF exposure n = 22	p
Median age [IQR] (years)	52 [45–63]	52 [46–57]	50 [44–58]	0.65
Male, n (%)	21 (75.0)	29 (93.5)	15 (68.2)	0.05
Mean bodyweight (SD) (kg)	63(10)	66 (12)	67 (13)	0.36
AIDS stage, n (%)	10 (35.7)	13 (41.9)	13 (59.1)	0.24
HIV viral load <20 copies/ml, n (%)	21 (77.8)	21 (67.7)	17 (77.3)	0.62
CD4 <200 cells/μL, n (%)	4 (14.3)	3 (9.7)	5 (22.7)	0.42
Anti-HCV+, n (%)	5 (17.9)	2 (6.4)	6 (27.3)	0.12
Arterial hypertension, n (%)	4 (14.3)	4 (12.9)	2 (9.1)	0.85
Diabetes, n (%)	1 (3.6)	1 (3.2)	3 (13.6)	0.23
Protease inhibitor/ritonavir, n (%)	22 (78.6)	20 (64.5)	16 (72.7)	0.48/0.23*
Median duration of TDF therapy [IQ] (months)	48 [40–67]	46 [21–69]	6.5 [0–37]	<0.001/0.43*
Median uPCR [IQR] (mg/g)	322.8 [260.6–445.6]	255.8 [221.5–335.2]	277.9[221.2–355.8]	0.10/0.03*
Median uAPR [IQR]	0.16 [0.11–0.24]	0.22 [0.19–0.31]	0.23 [0.13–0.33]	0.27
Median eGFR, [IQR] (ml/min/1.73m^2^)	84.1 [77.8–93.9]	104.6 [97.3–112.8]	84.8 [71.8–100.4]	<0.001

*comparison between the three groups / comparison TDF discontinuation group versus TDF continuation group

IQR, Interquartile range; SD, Standard deviation; uPCR, Urine protein-to-creatinine ratio; uAPR, Urine albumin-to-protein ratio

Patients who ceased TDF had, at baseline, higher median uPCR and lower eGFR than patients who continued TDF.

### Evolution of proteinuria

#### Persistence of tubular proteinuria

Fifteen of 75 (20%) patients followed for at least two years had a stable persistence of tubular proteinuria (presence at all visits up to 2 years, with an authorized exception): 4 patients were in the group "TDF discontinuation", 9 patients were in the group "TDF continuation" and 2 patients were in the group "no TDF exposure". Factors independently associated with persistence of tubular proteinuria were the continuation of TDF therapy [OR 9.0; 95% confidence interval (CI): 1.9;41.4; p = 0.01], and a higher level of proteinuria at baseline (> median baseline value (290 mg/g)) [OR 6.0; 95% CI: 1.3;28.0; p = 0.02].

#### Evolution of quantitative proteinuria

**Fig 1:**
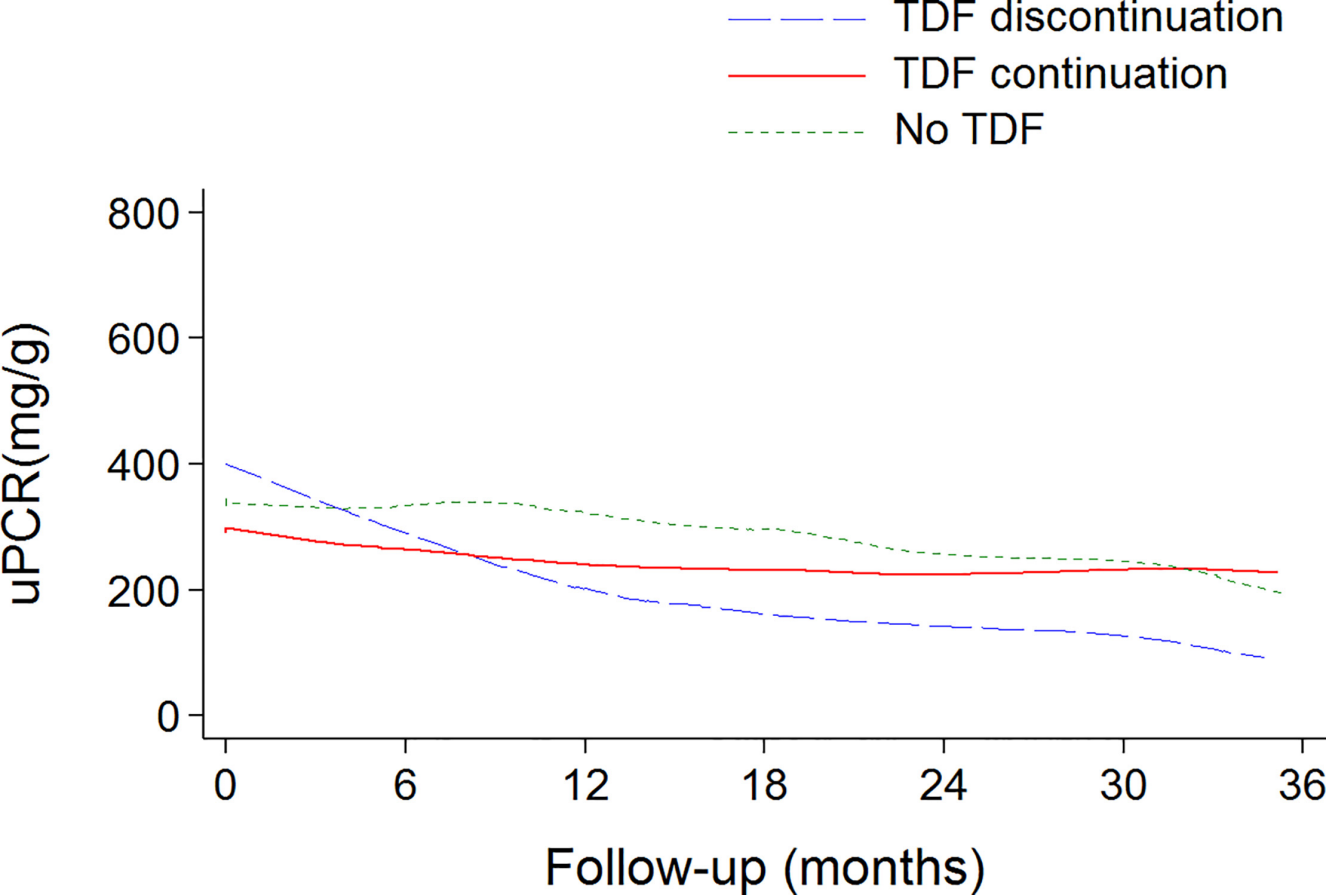
Evolution of uPCR during the follow-up, according to the TDF group. The solid lines denote lowess smoothing curves (regression-modelled values) summarizing the trends.

Overall uPCR decreased over time (mean slope: -4.4 mg/g/month; CI: -5.8;-3.8; P<0.001) and older age was associated with higher level of uPCR (3.8 mg/g per one-year increase; 95% CI:1.1;6.5; P = 0.004) (Fig 1). The comparison of the rate of decline between groups of TDF exposure indicated that uPCR decreased more rapidly in patients who discontinued TDF therapy than in patients who continued TDF and in those who did not receive TDF during the follow-up (Table 2).

**Table 2:** Evolution of quantitative proteinuria and eGFR by TDF exposure group.

	eGFR^a^ (mL/min/1.73m^2^/year)			uPCR^b^ (mg/g/month)		
	Mean slope	95% confidence interval	P^c^	Mean slope	95% confidence interval	P^c^
TDF discontinuation	-2.04	-3.69;-0.40	Ref	-7.7	-10.0;-5.3	Ref
TDF continuation	-3.77	-5.14;-2.39	0.09	-3.3	-5.2;-1.3	<0.001
No TDF exposure	-2.39	-4.26;-0.51	0.78	-3.8	-6.5;-1.2	0.004

^a^The model also includes age, sex, AIDS stage, undetectable viral load and boosted protease inhibitor exposure

^b^The model also includes age, sex, AIDS stage, positive hepatitis C serology and boosted protease inhibitor exposure

^c^P value for comparison of slopes of the groups “TDF continuation” and “no TDF exposure groups” with the group “TDF discontinuation” taken as the reference.

In subjects who discontinued TDF, the median uPCR level decreased from 297 mg/g at the time of discontinuation to 91 mg/g at the end of follow-up (Table 3).

**Table 3:** Levels of eGFR and uPCR at baseline, at the time of discontinuation and at the end of follow-up by TDF exposure.

	Baseline	Time of discontinuation	End of follow-up
Median uPCR [IQR] (mg/g)			
TDF discontinuation	323 [260–446]	297 [233–445]	91 [82–130]
TDF continuation	256 [221–335]		157 [120–197]
No TDF exposure	278 [221–356]		179 [111–368]
Median eGFR [IQR] (ml/min/1.73^2^)			
TDF discontinuation	84 [78–94]	83 [79–99]	82 [65–98]
TDF continuation	105 [97–113]		95 [78–105]
No TDF exposure	85 [72–100]		75 [60–94]

Of the 23 patients who discontinued TDF before 24 months of follow-up, 11 had uPCR below the threshold value of 200 mg/g at all follow-up visits consecutive to discontinuation of TDF.

### Evolution of eGFR

**Fig 2:**
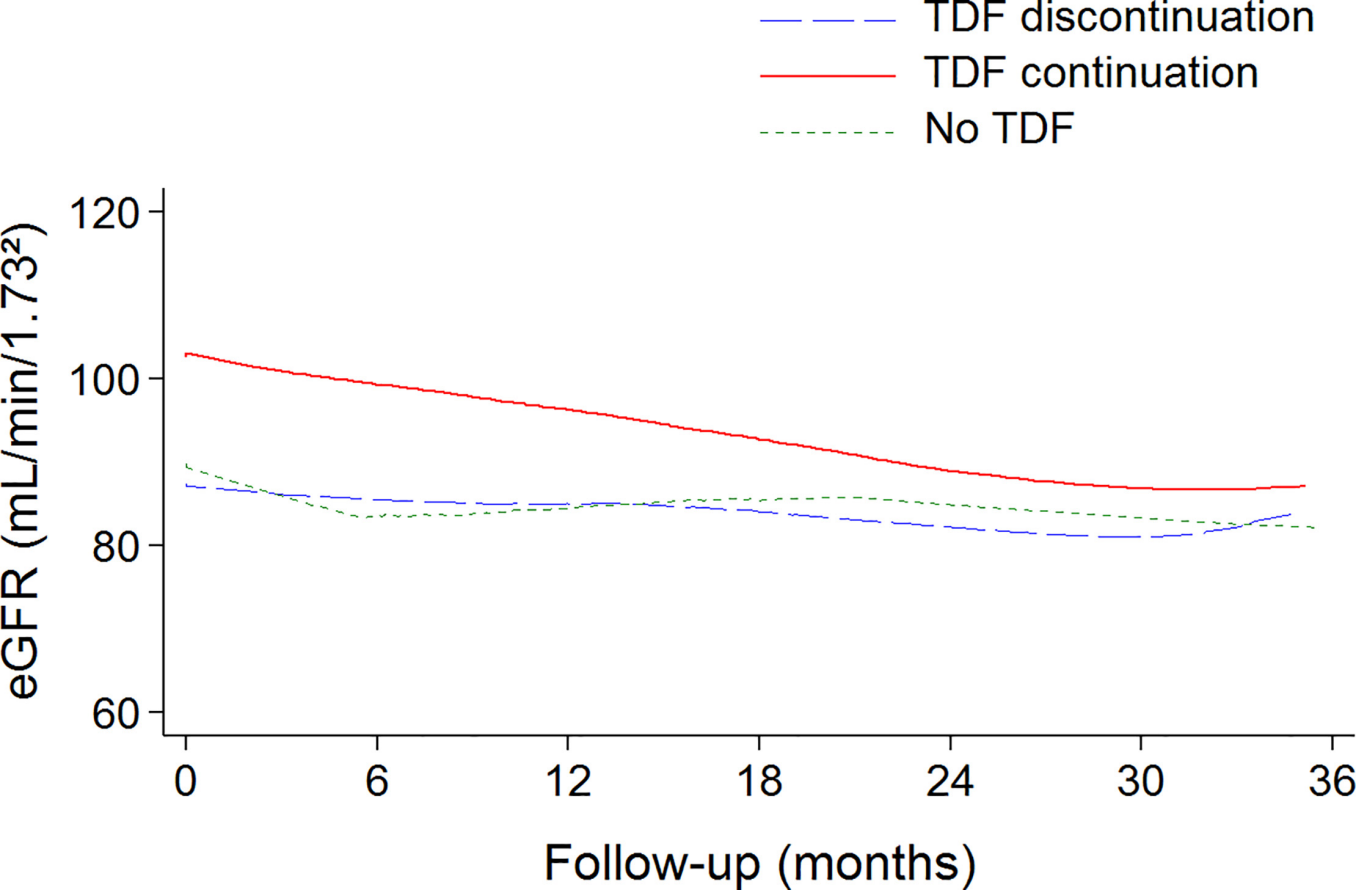
Evolution of eGFR during the follow-up, according to the TDF group. The solid lines denote lowess smoothing curves (regression-modelled values) summarizing the trends.

Overall, eGFR decreased over time with a mean rate of decline of -3.8 mL/min/1.73m^2^/year (95% CI: -4.6;-3.0; P<0.001) (Fig 2). Older age was associated with lower level of eGFR (-0.95 mL/min/1.73m^2^ per one-year increase (95% CI: -1.29; -0.62; P<0.001)). Mean level of eGFR during the follow-up was higher in patients who continued than in those who discontinued TDF (difference: 15.4 mL/min/1.73m^2^; 95% CI: 7.3;23.4), but the rate of decline over time tended to be higher in those who continued than in those who discontinued TDF (Table 2). The discontinuation of TDF was associated with a reduction in the eGFR decline (-5.8 mL/min/1.73m^2^/year; 95% CI: -8.2;-3.2 during TDF exposure versus -3.0 mL/min/1.73m^2^/year; 95% CI: -4.2;-1.8 after TDF discontinuation; P = 0.01).

## Discussion

The aim of this study was to describe the evolution of renal dysfunction and evaluate the impact of TDF discontinuation in patients with eGFR ≥ 60 ml/min/1.73 m^2^, detected with tubular proteinuria at baseline. After two years of follow-up, 20% of patients had stable persistence of tubular proteinuria. Continuation of TDF was the main factor associated with this persistence.

In our study, TDF was stopped in 28 patients, mainly for renal abnormalities or prevention of renal function worsening, and after a median follow-up of 7.5 months. At baseline, these patients had higher uPCR and lower eGFR compared to patients who continued TDF. The previous study [11], by detecting the mild renal impairment, has attracted the attention of physicians to patients, increased alertness and resulted in an early discontinuation of TDF to prevent worsening of kidney disease.

Overall uPCR decreased over time and data indicated that uPCR decreased more rapidly in patients who discontinued TDF than in patients who continued TDF (Fig 1). Moreover, the discontinuation of TDF was associated with a significant reduction in uPCR, and in 48% of patients who discontinued TDF, uPCR returned below the threshold of 200 mg/g. The normalization of proteinuria after TDF discontinuation is poorly documented. In a recent study, proteinuria significantly reduced in 11/12 patients who stopped TDF, because of proteinuria [10]. In another study, risk of kidney disease did not appear to decrease after TDF cessation [4]. In our study, in patients who continued TDF, uPCR decreased more slowly; however proteinuria did not worsen during the follow-up in these patients. Comparable data have been found in another recent study with a shorter follow-up. In this study, proteinuria did not significantly change in the 30 patients continuing TDF, over a six-month period [10].

In our study, overall, eGFR decreased over time with a mean annual rate of decline of 3.84 ml/min/1.73m^2^. In the general population, the mean rate of age-related decline in GFR is 0.75–1.00 ml/min per 1.73m^2^ every year after the age of 40 years [16]. A decline of more than 3–4 ml/min/year is considered as fast [17].

Our results suggested that discontinuation of TDF was associated with a slower decline in eGFR. In a study, TDF use was associated with an 11% increased risk of rapid decline per year of exposure [4]. Another recent study found a rapid decline in eGFR during the first 3 months of TDF therapy (mean slope -15.7 ml/min/1.73m^2/^year; 95% CI -20.5 to -10.9), and a slighter decline thereafter (mean slope -3.1 ml/min/1.73m^2^/year; 95% CI -4.6 to -1.7) [7].

Several studies have assessed the reversibility of eGFR after cessation of TDF. In a study, 22 months after TDF discontinuation, renal parameters returned to normal values in 59% of patients, improved in 9.8%, and did not improve in 31% of cases [8]. In another study, 42% of patients who ceased TDF had a reversibility of eGFR [9]. In a more recent study, decline in the eGFR during TDF therapy was not fully reversible in one third of patients [7].

Our study has some limitations. First, our study concerns a small number of patients, which may lead to a lack of power. Secondly, the detection of the initial proteinuria was made with only one urinary sample, so we cannot rule out that the initial proteinuria was a transitory event. Thirdly, our study was observational, and the decision to stop TDF was made by each physician based on the evolution of the patient's condition without collegial decision or preexistent recommendations.

In our patients, after two years of follow-up, 20% of patients had a stable persistence of tubular proteinuria, TDF being the main associated factor. Although continuation of TDF was not associated with worsening of proteinuria, a normalization of proteinuria was observed in only 48% of patients who discontinued TDF. However, the clinical significance of this TDF-related low level of proteinuria as a factor associated with renal disease progression and bone loss is poorly understood. The early detection and regular follow-up of renal dysfunction in HIV-infected adults and more precisely in those treated with TDF have to be considered.
